# Pyranose Dehydrogenase Ligand Promiscuity: A Generalized Approach to Simulate Monosaccharide Solvation, Binding, and Product Formation

**DOI:** 10.1371/journal.pcbi.1003995

**Published:** 2014-12-11

**Authors:** Michael M. H. Graf, Lin Zhixiong, Urban Bren, Dietmar Haltrich, Wilfred F. van Gunsteren, Chris Oostenbrink

**Affiliations:** 1 Food Biotechnology Laboratory, Department of Food Science and Technology, University of Natural Resources and Life Sciences (BOKU), Vienna, Austria; 2 Laboratory of Physical Chemistry, Swiss Federal Institute of Technology, ETH, Zürich, Switzerland; 3 Institute of Molecular Modeling and Simulation, University of Natural Resources and Life Sciences (BOKU), Vienna, Austria; 4 Laboratory for Molecular Modeling, National Institute of Chemistry, Ljubljana, Slovenia; University of Maryland, United States of America

## Abstract

The flavoenzyme pyranose dehydrogenase (PDH) from the litter decomposing fungus *Agaricus meleagris* oxidizes many different carbohydrates occurring during lignin degradation. This promiscuous substrate specificity makes PDH a promising catalyst for bioelectrochemical applications. A generalized approach to simulate all 32 possible aldohexopyranoses in the course of one or a few molecular dynamics (MD) simulations is reported. Free energy calculations according to the one-step perturbation (OSP) method revealed the solvation free energies (ΔG_solv_) of all 32 aldohexopyranoses in water, which have not yet been reported in the literature. The free energy difference between β- and α-anomers (ΔG_β-α_) of all d-stereoisomers in water were compared to experimental values with a good agreement. Moreover, the free-energy differences (ΔG) of the 32 stereoisomers bound to PDH in two different poses were calculated from MD simulations. The relative binding free energies (ΔΔG_bind_) were calculated and, where available, compared to experimental values, approximated from *K*_m_ values. The agreement was very good for one of the poses, in which the sugars are positioned in the active site for oxidation at C1 or C2. Distance analysis between hydrogens of the monosaccharide and the reactive N5-atom of the flavin adenine dinucleotide (FAD) revealed that oxidation is possible at HC1 or HC2 for pose A, and at HC3 or HC4 for pose B. Experimentally detected oxidation products could be rationalized for the majority of monosaccharides by combining ΔΔG_bind_ and a reweighted distance analysis. Furthermore, several oxidation products were predicted for sugars that have not yet been tested experimentally, directing further analyses. This study rationalizes the relationship between binding free energies and substrate promiscuity in PDH, providing novel insights for its applicability in bioelectrochemistry. The results suggest that a similar approach could be applied to study promiscuity of other enzymes.

## Author Summary

Generally, enzymes are perceived as being specific for both their substrates and the reaction they catalyze. This standard paradigm started to shift and currently enzyme promiscuity towards various substrates is perceived rather as the rule than the exception. Enzyme promiscuity seems to be vital for proteins to acquire new functions, and therefore for evolution itself. The driving forces for promiscuity are manifold and consequently challenging to study. Binding free energies, which can be calculated from computer simulations, represent a convenient measure for them. Here, we investigate the binding free energies between the enzyme pyranose dehydrogenase (PDH) and a sugar-substrate by computational means. PDH has an extraordinarily promiscuous substrate-specificity, making it interesting for *e.g.* bioelectrochemical applications. By introducing modifications to the sugar-structure used for the molecular dynamics simulations, we could simultaneously study all 32 possible aldohexopyranoses from a single simulation. This saves costly computational resources and time for setting up and analyzing the simulations. We could nicely reproduce experimental results and predict so far undetected sugar-oxidation products, directing further experiments. This study gives novel insights into PDH's substrate promiscuity and the enzyme's applicability. A similar approach could be applied to study the promiscuity of other enzymes.

## Introduction

Generally, enzymes are perceived as being specific for both their substrates and the reactions they catalyze [1]. Deviations from such behavior are often seen as unwanted side effects or even errors in the biological function of the enzyme that come at an additional energetic cost for the organism. Although this feature has long been recognized to be useful in other contexts, for example in the recognition of multiple antigens by the same germline antibody [2]–[4], such enzymes are often characterized by poor overall catalytic efficiencies and termed promiscuous. Starting in 1976, this paradigm started to shift when Jensen drew a link between promiscuity and protein evolution [5]. He hypothesized that the first enzymes had very broad substrate specificities that evolved to more specialized forms via duplication, mutation, and selection of the corresponding genes. This was corroborated by later studies that investigated the evolutionary implications of promiscuity such as the adaption of enzymes towards novel xenobiotics, e.g. halogenated compounds or antibiotics, in the course of a few decades [6], [7]. Although systematic screens for promiscuous enzyme functions are not feasible because of the vast number of possible different substrates and reactions, there are many indications and examples that promiscuity is rather the rule than the exception [6]. Especially in the past two decades, enzyme promiscuity received considerable attention, and enzymes that can take over the function of related enzymes in an organism *via* their promiscuous activities have been extensively investigated [8]–[10]. These studies suggest that metabolic pathways are intertwined in many unexpected ways, which ultimately gives the organism a higher survival potential under changing environmental conditions. Regulation of such metabolic pathways as well as promiscuity itself at the gene-, transcript-, protein-, and localization-level and the associated reaction conditions are other thriving research areas [1], [11]. Moreover, promiscuity is often observed for close homologs in protein families and distant homologs within superfamilies [11]. Individual family members have frequently evolved from a common ancestor through gene duplication and subsequent specialization. These members share the same fold and catalytic strategy, and consequently the main activity of one family member is often found as the promiscuous activity of another family member. Nobeli and coworkers refer to this phenomenon as ‘family’ promiscuity as opposed to ‘individual’ or ‘pure’ promiscuity, which is associated to multiple functions of a single enzyme [11]. The molecular mechanisms underlying promiscuity are manifold, including post-translational modifications, multiple domains, oligomeric states, protein flexibility, partial recognition, multiple interaction sites or a single site with diverse interacting residues, allosteric interactions, flexibility as well as size and complexity of the interaction partner, chemical scaffolds, and protonation states of active site residues [6], [11], [12]. Hydrophobic interactions, diverse hydrogen bonding, flexibility, and nonpolar van der Waals interactions combined with negligible electrostatics were found to be the main driving forces for promiscuity [11], [13]–[15]. Consequently, understanding the molecular mechanisms and energetics leading to enzyme promiscuity is a valuable asset in the field of protein design and engineering as well as drug development, and therefore they have been investigated extensively [1], [16]. In view of various causes and effects involving promiscuity, it is not surprising that the definition of the term is not exact and combinations of different definitions occur [1], [6], [11], [16], [17]. In this article, the term ‘promiscuity’ is used in the context of relaxed substrate specificity [1], [18] in order to perform similar chemical reactions on related substrates [17].

A prototypical example of ‘family’ promiscuity [11] can be found in the structural family of GMC oxidoreductases, named after three representatives utilizing either glucose, methanol, or choline as their substrate [19]. Although the four initially characterized GMC family members share only between 23 and 32% sequence similarity and possess diverse catalytic activities with a wide range of substrate specificity, they share the same overall architecture and catalyze a similar chemical reaction, the oxidation of an alcohol moiety. Cavener speculates about an ancestral protein of this family that could bind to many different substrates [19], which it converted with low catalytic efficiencies while mutations paved the way for high specificities towards the individual substrates. A more recent addition to the GMC oxidoreductase family is the glycosylated enzyme pyranose dehydrogenase (PDH, EC 1.1.99.29), reacting with many different carbohydrates. It contains a monocovalently bound flavin adenine dinucleotide (FAD) and has a mass of approximately 65 kDa. Although PDHs from other sources with similar biochemical properties have been studied [20]–[22], the enzyme from *Agaricus meleagris* has been characterized most extensively so far: a wealth of biochemical data [13], [23]–[25] and a high-resolution X-ray structure with PDB code 4H7U [26] are readily available. To date, its exact physiological role is unknown. However, because of the natural habitat of *Agaricus meleagris* on lignocellulose-rich forest litter and PDH's reactivity with a multitude of different carbohydrates found during lignin degradation, PDH is most likely involved in lignocellulose breakdown [25]. Compared to other GMC oxidoreductases, it oxidizes many different aldoses and ketoses in pyranose form as well as heteroglycosides, glucooligosaccharides, sucrose, and lactose, which can be (di)oxidized at C1–C4. A comprehensive list of its impressively broad substrate spectrum can be found in the paper of Sedmera and coworkers [27] and an updated version in the review of Peterbauer and Volc [25]. The reactivity towards many different carbohydrate-substrates makes PDH a very interesting enzyme to study in the context of substrate-promiscuity. In this manuscript, we investigate computationally the promiscuous nature of PDH towards the pyranose form of monosaccharides that are turned over by the enzyme. All 32 possible combinations of α- and β-anomers as well as d- and l-stereoisomers of glucose, mannose, galactose, talose, allose, altrose, gulose, and idose will be considered.

Molecular dynamics (MD) simulations of PDH were applied to study the interactions with the monosaccharides described above. The aim of this study was to gain a deeper understanding of the promiscuous nature of PDH towards monosaccharides. This involved a generalized approach of extensive MD simulations and free energy calculations using the one-step perturbation (OSP) method [28], [29] to calculate monosaccharide binding and solvation. The OSP method is an efficient means to obtain free-energy differences of similar molecules from a simulation of a carefully designed reference molecule for which the sampling is such that configurations are sampled that are representative of the molecules or states in which one is interested. In the past, OSP has successfully been applied to reproduce and predict binding free energies of a series of compounds to a common receptor [29], [30], to study the stereoselective binding of substrates to a promiscuous enzyme [31], [32], to study conformational preferences of molecules that show slow transitions in regular simulations [33], [34] or the effect of changes in force-field parameters on conformational equilibria [35], [36].

In the current work, we investigate (i) the solvation free energies of the 32 above-mentioned monosaccharides in water; (ii) the free energy differences of the α/β-anomers of the d-stereoisomers in water; (iii) the relative binding free energies for all monosaccharides and (iv) the occurrence of reactive poses for all monosaccharides. Where experimental data were available, comparisons were made and good to excellent agreements were observed. Furthermore, our work offers predictions of properties that have not yet been described experimentally.

## Methods

### 2.1. Preparation of initial structures

**Figure 1:**
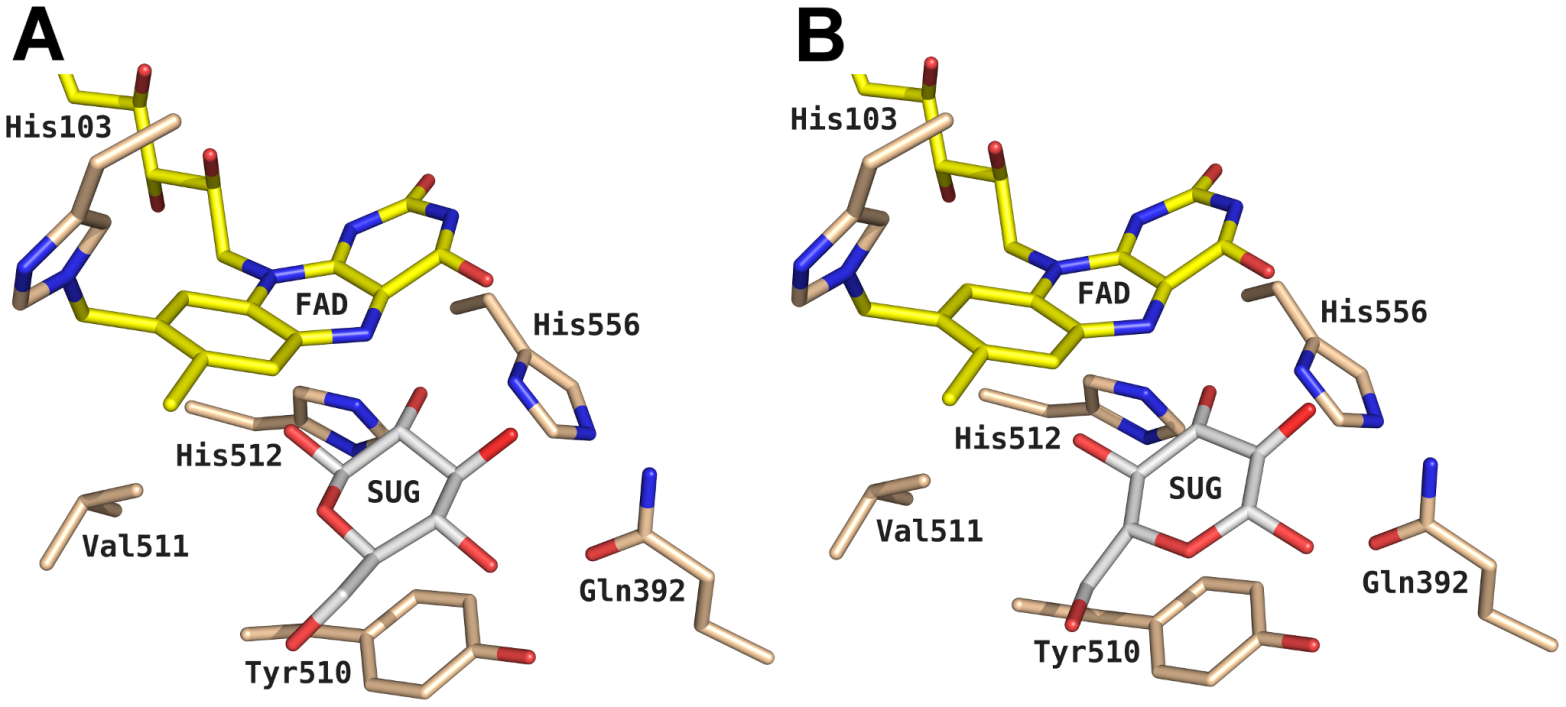
Active site of pyranose dehydrogenase (PDH; PDB code 4H7U) from *Agaricus meleagris* with bound sugar (SUG; here β-d-glucose). To transfer the sugar coordinates from pyranose 2-oxidase (P2O) from *Trametes multicolor* into PDH, the X-ray structures of both enzymes were first superimposed. The β-d-glucose coordinates from the P2O structures with PDB codes (A) 3PL8 – termed pose A – or (B) 2IGO – termed pose B – were grafted into PDH. Atom-coloring scheme: carbon (beige, protein; yellow, FAD; white, ligand), nitrogen (blue), and oxygen (red). The figure was generated using PyMOL (http://www.pymol.org/).

**Figure 2:**
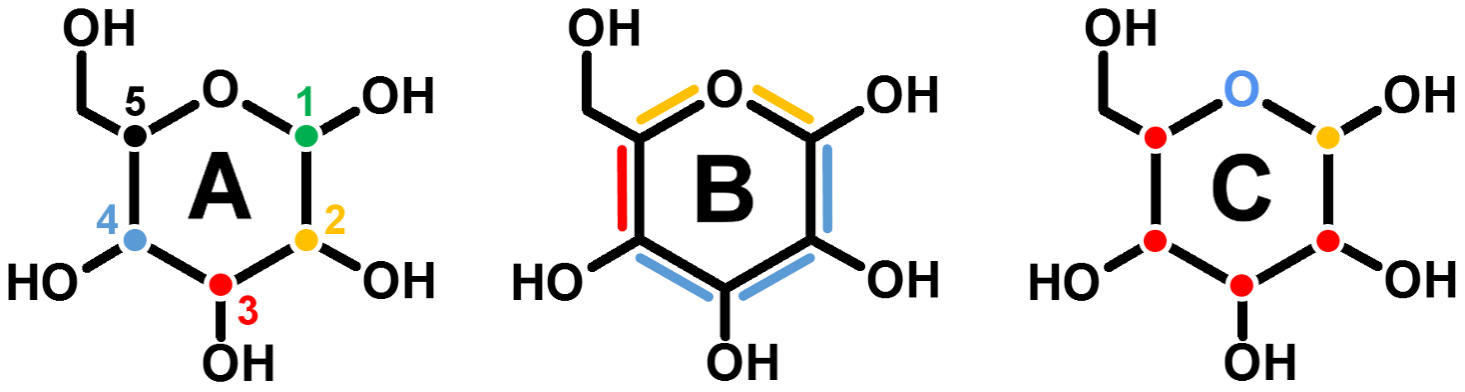
Modifications made to the topology of system SUG (also refer to Supplementary Data). (A) System SUG^a^: improper dihedrals (ID) at stereocenters C1–C5 were turned off. Numbers indicate the name of the C-atom and the ID-position within the 5-digit ID code in Tables 1 and 3. Colors are in agreement with the coloring schemes in Figs. 5–7: green (C1, ID1), yellow (C2, ID2), red (C3, ID3), blue (C4, ID4), and black (C5, ID5). (B) System SUG^ab^: in addition to system SUG^a^, proper dihedral force constants (*k_φ_*) for the ring torsional dihedral angles were lowered according to the following coloring scheme: blue (two force constants from 2.09 to 0.418 kJ mol^−1^ and one from 5.92 to 1.05 kJ mol^−1^), red (one force constant from 5.92 to 1.05 kJ mol^−1^), and yellow (two force constants from 3.77 to 1.05 kJ mol^−1^). (C) System SUG^abc^: in addition to system SUG^ab^, bond angle bending force constants (*k_θ_*) for the bond angles surrounding the ring atoms (C1–C5 and O) were lowered according to following coloring scheme: blue (one bond angle from 380 to 285 kJ mol^−1^), red (two bond angles from 320 to 285 kJ mol^−1^), yellow (three bond angles from 320 to 285 kJ mol^−1^).

The structure preparations were essentially performed as reported previously [13]. In short, a preliminary version of the 1.6 Å resolution X-ray structure of PDH (PDB code 4H7U) served as starting point [26]. The covalent monoatomic oxygen species at C(4a), which is most likely an X-ray artifact, was removed. As a glycoprotein, the structure of PDH comprised covalently attached sugar moieties at surface residues Asn-75 and Asn-294. The influence of these glycosylated residues on the active site is expected to be negligible and consequently the glycan structures were removed. A PO_4_^3-^ ion at the surface, which is most likely a crystallization buffer artifact, was removed as well. The amino and carboxy termini were charged; all arginines, cysteines and lysines were protonated, and all aspartates and glutamates were deprotonated. In our previous study, we propose that PDH oxidizes its sugar substrate *via* a general base proton abstraction [13], which requires one of the two active site histidines (His-512 and His-556) being neutrally charged. The most stable protonation state fulfilling this requirement was obtained when His-512 was fully protonated and His-556 was in its neutral state (proton at N_ε_). The selection of the tautomeric state for the neutral His-556 was such that in the X-ray structure its deprotonated nitrogen atom pointed towards the active site. The remaining histidines were doubly protonated, except for His-103, which is covalently attached to the FAD and was protonated at N_δ_. No structure of PDH comprising a monosaccharide-substrate in the active site was available at commencement of this work. Therefore, PDH and the closely related GMC oxidoreductase pyranose 2-oxidase (P2O, EC 1.1.3.10) were superimposed with an atom-positional root-mean-square deviation (RMSD) of 0.13 nm for all heavy atoms of their sugar-binding sites. Two different P2O structures were used, in which the bound sugar roughly differs in a 180° rotation around the axis going through C2 and C5 of the tetrahydropyrane ring to allow for (di)oxidations at all possible sites (C1–C4). Superposition of PDH and P2O in complex with 3-fluoro-3-deoxy-β-d-glucose (PDB: 3PL8) [37] yielded pose A (Fig. 1A), whereas pose B (Fig. 1B) was obtained by aligning PDH with P2O in complex with 2-fluoro-2-deoxy-β-d-glucose (PDB: 2IGO) [38]. After grafting the monosaccharide coordinates into PDH's active site, the fluorine of the sugar was replaced by a hydroxyl group. This procedure ultimately yielded system PDH-SUG, with the monosaccharide bound to PDH according to pose A or pose B. For simulations of sugar without PDH, the coordinates of β-d-glucose from P2O-PDB 2IGO [38] were used. For the description of the interactions with the sugar, a united atom force field was used. Chirality around CH-groups in such a force field is enforced through an improper dihedral potential energy term. In order to allow transitions between equatorial and axial positions of the attached hydroxyl groups and to sample all 32 possible monosaccharides in a single MD simulation, following changes were made to the topology of β-d-glucose, following suggestions in references [28] and [31] as indicated in Fig. 2:

- Improper dihedral angle interactions at C1–C5 were turned off (Fig. 2A) – leading to model SUG^a^.
- In addition to (a), the proper dihedral force constants (*k_φ_*) for the ring torsional dihedral angles were lowered according to the coloring scheme in Fig. 2B – leading to model SUG^ab^.
- In addition to (a) and (b), the bond angle bending force constants (*k_θ_*) for the bond angles surrounding all ring atoms (C1–C5 and O) were lowered according to the coloring scheme in Fig. 2C – leading to model SUG^abc^.

The building block and the changes made to the β-d-glucose topology to define the three reference molecules are further detailed in the supplementary material (Table S1).

### 2.2. Simulation setup

MD simulations were conducted using the GROMOS 11 software package [39] employing the 53A6 force field [40]. In this parameter set, carbohydrates are described according to the parameter set of [41], and the topology of β-d-glucose is given in the supplementary material. Note that the GROMOS force field is a united atom force field, which is crucial for the modifications of the sugar interactions described in the previous section. His-103 and FAD were covalently attached to each other and their topologies and force field parameters were adapted accordingly. All systems were energy-minimized in vacuo employing the steepest-descent algorithm: for the PDH-SUG complexes, the sugar atoms were energy-minimized with constrained PDH coordinates after which both SUG and PDH atoms were energy-minimized. A 1-µs stochastic dynamics (SD) simulation of SUG in vacuum was performed, referred to as SUG_vac_, for which the energy-minimized structure of SUG was used. For MD simulations of SUG and the complex PDH-SUG in water (SUG_water_ and PDH-SUG, respectively) the structures were placed into a rectangular, periodic, and pre-equilibrated box of SPC water [42]. All water molecules within 0.23 nm of any solute atom were removed from the box and a minimum solute to box-wall distance of 0.8 nm was enforced. In order to relax unfavorable atom-atom contacts between the solute and the solvent, energy-minimization of the solvent was performed while keeping the solute positionally restrained using the steepest-descent algorithm. Finally, five water molecules with the most favorable electrostatic potential for replacement by a positive ion were substituted with sodium ions to achieve electroneutrality in systems PDH-SUG.

The following protocol was used to thermalize and equilibrate the system: initial velocities were randomly assigned according to a Maxwell-Boltzmann distribution at 50 K. All solute atoms were positionally restrained through a harmonic potential energy term with a force constant of 2.5×10^4^ kJ mol^−1^ nm^−2^ in order not to disrupt the initial conformation, and the systems were propagated for 20 ps. In five subsequent 20 ps MD simulations, the positional restraints were reduced by one order of magnitude and the temperature was increased by 50 K. Subsequently, the positional restraints were removed, roto-translational constraints introduced on all solute atoms [43], and the systems were further equilibrated, each for 20 ps at 300 K. Finally, an equilibration at a constant pressure of 1 atm was conducted for 300 ps.

After equilibration, production runs at constant pressure (1 atm) and temperature (300 K) were performed. For the SUG_water_ systems, one production run of 100 ns was performed. For the PDH-SUG systems, each with SUG^a^, SUG^ab^, or SUG^abc^ bound according to pose A or pose B, two independent 50 ns production runs (termed md1 and md2) were conducted, leading to a total of 12 independent PDH-SUG simulations. Pressure and temperature were kept constant using the weak-coupling scheme [44] with coupling times of 0.5 and 0.1 ps, respectively. The isothermal compressibility was set to 4.575×10^−4^ kJ^−1^ mol nm^3^, and two separate temperature baths were used for solute and solvent. The SHAKE algorithm was applied to constrain all solute bond lengths [45] as well as the solvent geometry in simulation SUG_water_. Because of simulation efficiency, the SETTLE algorithm was applied to constrain solvent geometry [46] in system PDH-SUG. In all cases, constraining the bond lengths allowed for 2-fs time-steps. Nonbonded interactions were calculated according to a triple range scheme. Interactions within a short-range cutoff of 0.8 nm were calculated at every time-step from a pair list that was updated every fifth step. At these points, interactions between 0.8 and 1.4 nm were also calculated explicitly and kept constant between updates. A reaction field [47] contribution was added to the electrostatic interactions and forces to account for a homogenous medium outside the long-range cutoff using a relative dielectric constant of 61 as appropriate for the SPC water model [48]. Coordinate and energy trajectories were stored every 0.5 ps for subsequent analysis.

### 2.3. Free energy calculations

The one-step perturbation (OSP) method relies on the application of Zwanzig's perturbation formula which is exact in the limit of infinite sampling [49]. In practice, the free-energy difference between a possibly unphysical reference molecule represented by Hamiltonian 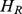 and a physically relevant compound represented by Hamiltonian 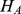 is accurately estimated if a simulation of the reference molecule samples a sufficiently high number of configurations relevant for the real compound. In those cases the free energy can efficiently be calculated using
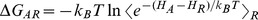
(1)where the angular brackets 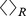 indicate an ensemble average computed from the simulation of the reference state and 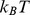 represents Boltzmann's constant multiplied by the absolute temperature. Since only the energy difference 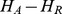 appears in the exponential, only the few energy terms that are different between the compounds need to be re-evaluated over the real compounds, here involving only the covalent interactions indicated in Fig. 2.

The free energy differences 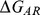 can subsequently be used to estimate various physically relevant free energy differences, such as the solvation free energies, relative to the reference state
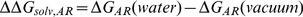
(2)


The free energy difference between α- and β-anomers of specific sugars can be computed as
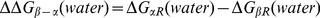
(3)where the subscripts α and β refer to the α- and β-anomers of a single monosaccharide. The binding free energy relative to the reference compound is calculated as
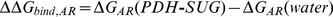
(4)and relative to another compound as
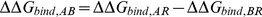
(5)


One limitation of the OSP approach is the fact that most simulation effort is spent on unphysical reference molecules, reducing the direct insight into the structure and dynamics of the real compounds. However, the ensemble average of any property Q for the real compounds may be obtained using [50], [51]

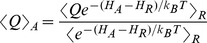
(6)which was used here to analyze the average occurrence of reactive poses for the real compounds. The distances between H-atoms HC1, HC2, HC3, and HC4 and the N5 atom of the FAD cofactor were calculated for the reference state simulations as 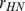. Consistent with our previous study [13], a particular conformation was considered as reactive for a specific carbon if the corresponding value of 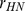 was below 0.3 nm, such that the average occurrence can be calculated as
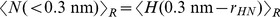
(7)where H(x) is the Heaviside step function, *i.e.* 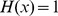 for 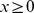 and 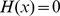 for 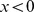. By replacing Q in equation (6) by 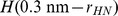, we obtain the average amount of catalytically active poses of the real compounds.

In the current work, multiple reference compounds R were applied (SUG^a^, SUG^ab^, and SUG^abc^) whereas individual estimates were combined by transferring the free energy estimates to a common reference state. One can easily show that expressing the ensemble average for reference compound R1 of equation (1) as an umbrella-weighted ensemble, calculated from a simulation of reference state R2 using equation (6), can be expressed as
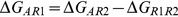
(8)where both terms on the right-hand side are readily calculated from the simulation of R2. This way, simulations of the three reference states lead to three estimates of 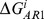, which can be exponentially averaged to obtain
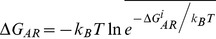
(9)where the overbar indicates an average over three values of i [52]. Statistical error estimates for the individual ensemble averages used in equation (1) were obtained from covariances and the statistical inefficiency as described in [53]. The uncertainty in a series of N correlated sequential observations *x_n_*, with expectation value 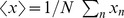, becomes 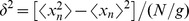 where *g* is the statistical inefficiency, defined as *g* = 1+2*τ*, with *τ* the auto-correlation time of the normalized autocorrelation function, 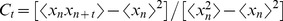 [53]. The individual error estimates of 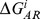 were subsequently weighted by 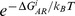 to obtain the statistical uncertainty on 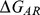.

## Results/Discussion

### 3.1. Monosaccharide solvation

**Figure 3:**
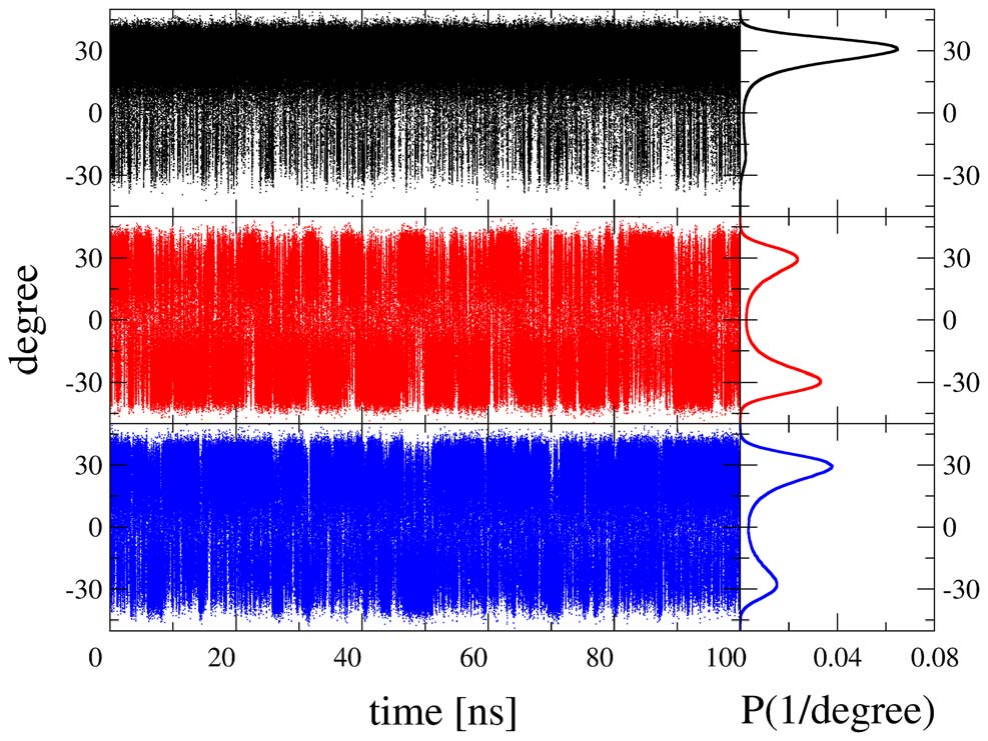
Distributions of the improper dihedral 5 for the three 100 ns MD simulations of system SUG_water_ with changes to the topology according to Fig. 2. Coloring scheme: SUG^a^ (black), SUG^ab^ (red), and SUG^abc^ (blue).

To find a suitable reference state, which is crucial for reliable free energy calculations according to the OSP method, MD simulations of system SUG_water_ with changes to the topology according to SUG^a^, SUG^ab^, and SUG^abc^ were conducted. As a typical example, Fig. 3 shows the distributions of the improper dihedral angle 5 (ID5) centered on atom C5 for the three 100 ns MD simulations. For 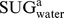 (black), ID5 is not evenly distributed and samples mostly the region around +30 degrees. 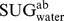 (red) and 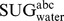 (blue) both show more equal distributions, indicating that both stereo-configurations are equally sampled. To use a topology with minimal changes with respect to the real compounds, 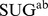 was selected as the most suitable reference state in water. Similarly, 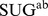 was used for the 1 µs SD simulation in vacuo (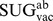). In contrast, SUG^a^, SUG^ab^, and SUG^abc^ (Fig. 2) were all selected for simulations and analysis of system PDH-SUG, in order to sample as many stereoisomers as possible. Consequently, 12×50 ns MD simulations of system PDH-SUG were conducted: three different SUG topologies, two different SUG binding poses (pose A and pose B) and two independent simulations for each (md1 and md2). MD simulations of system PDH-SUG will be referred to as *e.g.* 
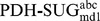
 (pose A).

Table 1 shows the 32 simulated stereoisomers, their 5-digit ID code, and the corresponding sugar names. For systems 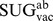 and 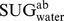, the relative free energies of individual stereoisomers with respect to the reference state in kJ/mol are listed. The range in relative free energies amounts to 13.6 kJ/mol (20.2–33.8 kJ/mol) for system 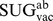 and to 18.7 kJ/mol (26.9–45.6 kJ/mol) for system 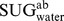. In achiral environments such as vacuum and water, no differences in the relative free energies are expected between enantiomers. In Table 1, sugar-pairs with codes 1 and 32, 2 and 31, 3 and 30, etc. represent enantiomers. Except for enantiomers β-d-talose (40.8 kJ/mol) and α-l-allose (45.6 kJ/mol) in system 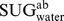, the relative free energies for the enantiomers match very well within both systems: absolute differences between 0.1–1.8 kJ/mol in system 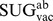 and 0.1–2.6 kJ/mol in system 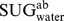 are roughly within the thermal noise of *k_B_T.* The relative free energies for β-d-talose and α-l-allose match qualitatively as they are the two largest within system 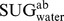. The value for α-l-allose seems exceptionally high and omission of this value reduces the range for system 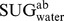 to 13.9 kJ/mol, similar to the vacuum value. Overall, the small free-energy differences between enantiomers give confidence in the applicability of the reference compound and in subsequent calculations. In the last column of Table 1, the calculated relative solvation free energies (ΔΔ*G_solv_*) are listed. To the best of our knowledge, values for these quantities have not been reported in the literature previously, neither from experimental nor from computational sources. While the values of ΔΔ*G_solv_* are relative to the reference state, the differences between these values provide insights into the solvation of monosaccharides.

**Table 1:** Relative free-energy differences (ΔG) of the SD or MD simulations for the 32 sugars with respect to the reference state in vacuum (SUG_vac_) or water (SUG_water_) and their calculated relative free energies of solvation (ΔΔG_solv_) in kJ/mol.

Sugar code	Improper dihedral code	Sugar name	ΔG [kJ/mol]		ΔΔG_solv_ [kJ/mol]
			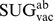	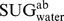	
1	22222	β-d-glucose	26.7±0.2	31.7±0.9	5.0±0.9
2	24222	β-d-mannose	32.4±0.3	37.9±1.0	5.6±1.0
3	22242	β-d-galactose	33.1±0.3	34.5±1.3	1.5±1.3
4	24242	β-d-talose	31.8±0.3	40.8±2.5	9.1±2.5
5	42222	α-d-glucose	20.5±0.1	34.4±1.0	13.9±1.0
6	44222	α-d-mannose	29.3±0.1	33.9±0.5	4.5±0.5
7	42242	α-d-galactose	25.3±0.1	36.2±1.2	10.9±1.2
8	44242	α-d-talose	30.1±0.1	37.8±0.5	7.7±0.5
9	22422	β-d-allose	24.8±0.1	26.9±0.4	2.1±0.4
10	24422	β-d-altrose	29.1±0.2	28.7±0.3	−0.5±0.4
11	22442	β-d-gulose	25.2±0.2	27.6±0.4	2.4±0.4
12	24442	β-d-idose	28.2±0.1	32.7±0.3	4.5±0.3
13	42422	α-d-allose	26.9±0.1	31.6±0.3	4.7±0.3
14	44422	α-d-altrose	29.4±0.1	29.7±0.4	0.3±0.4
15	42442	α-d-gulose	24.6±0.2	31.3±0.3	6.7±0.4
16	44442	α-d-idose	28.0±0.1	32.6±0.4	4.6±0.4
17	22224	β-l-glucose	29.1±0.1	30.8±0.2	1.7±0.2
18	24224	β-l-mannose	23.7±0.1	30.1±0.2	6.4±0.2
19	22244	β-l-galactose	29.8±0.1	28.0±0.2	−1.7±0.2
20	24244	β-l-talose	27.0±0.1	30.4±0.3	3.4±0.3
21	42224	α-l-glucose	27.5±0.2	31.2±0.2	3.6±0.3
22	44224	α-l-mannose	25.6±0.2	27.2±0.3	1.6±0.4
23	42244	α-l-galactose	29.0±0.2	27.0±0.2	−2.0±0.3
24	44244	α-l-talose	26.1±0.2	27.0±0.4	0.9±0.4
25	22424	β-l-allose	31.2±0.2	35.2±0.8	4.0±0.8
26	24424	β-l-altrose	23.6±0.1	34.6±0.9	10.9±0.9
27	22444	β-l-gulose	29.5±0.1	32.4±0.3	3.0±0.3
28	24444	β-l-idose	20.2±0.1	33.2±0.8	12.9±0.8
29	42424	α-l-allose	33.6±0.2	45.6±1.4	12.1±1.4
30	44424	α-l-altrose	33.8±0.4	36.2±1.4	2.4±1.5
31	42444	α-l-gulose	30.8±0.3	38.6±0.9	7.8±0.9
32	44444	α-l-idose	27.4±0.2	33.5±1.0	6.1±1.0

The digits ‘2’ or ‘4’ of the improper dihedral code in this table corresponds to the improper dihedral type code listed in the IMPDIHEDRAL-block of the SUG-topology (S1 Table). Note that the sequence of the digits ‘2’ and ‘4’ follows the improper dihedral angles of the sugar according to Fig. 2A.

Hydrolysis at the pyranose C1 atom allows for interconversion between the α- and β-anomers characterized by a corresponding equilibrium. Table 2 lists the free energy differences of β/α-anomers (ΔG_β−α_) of all simulated pyranose d-stereoisomers in water, for which comparison with the available experimental data is possible. The calculated values were obtained from equation (3), while the experimental values were calculated from previously published experimental estimates of the β/α-pyranose ratios. The experimental values (at 30°C) were found to be largely temperature-insensitive [54], and can be readily compared to the simulation data obtained at 300 K or 26.85°C. Estimates of ΔG_β−α_ from the experimental β/α-pyranose ratios were calculated according to
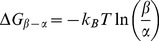
(10)


**Table 2:** Free energy differences of β/α-anomers of all simulated pyranose d-stereoisomers in kJ/mol for which experimental data are available.

Sugar	ΔG_β−α_ [kJ/mol]	
	Experiment [54]	Simulation
d-glucose	−1.2	−2.8
d-mannose	1.8	4.1
d-galactose	−1.7	−1.7
d-talose	1.0	3.0
d-allose	−4.1	−4.7
d-altrose	−1.1	−1.0
d-gulose	−4.8	−3.7
d-idose	−0.3	0.1

The values were obtained as differences of relative free energies of the two anomers calculated from the 100 ns simulation of system 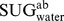 or from previously published experimental values [54].

The ΔG_β−α_ in Table 2 obtained from MD simulations or experiment have very small absolute deviations in a range between 0.0–2.3 kJ/mol, which is smaller than the thermal noise, with a mean absolute deviation of 0.5±1.3 kJ/mol.

For each of the 32 simulated monosaccharides of system 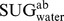, we investigated the occurrence of each of the 14 possible ring conformations [55], [56] of the six-membered pyranose ring by correlating the observed ring conformations with the values of the improper dihedral angles in simulation 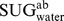. We found that sugars with code 1–16 (d-stereoisomers) occurred predominantly in the ^4^C_1_ chair conformation and sugars with code 17–32 (l-stereoisomers) in the ^1^C_4_ chair conformation. This again agrees with experimentally observed ring-conformational preferences of the d- or l-series of the studied aldohexopyranoses [55]–[57], which gives additional confidence in the conducted MD simulations.

### 3.2. Monosaccharide binding to PDH

**Figure 4:**
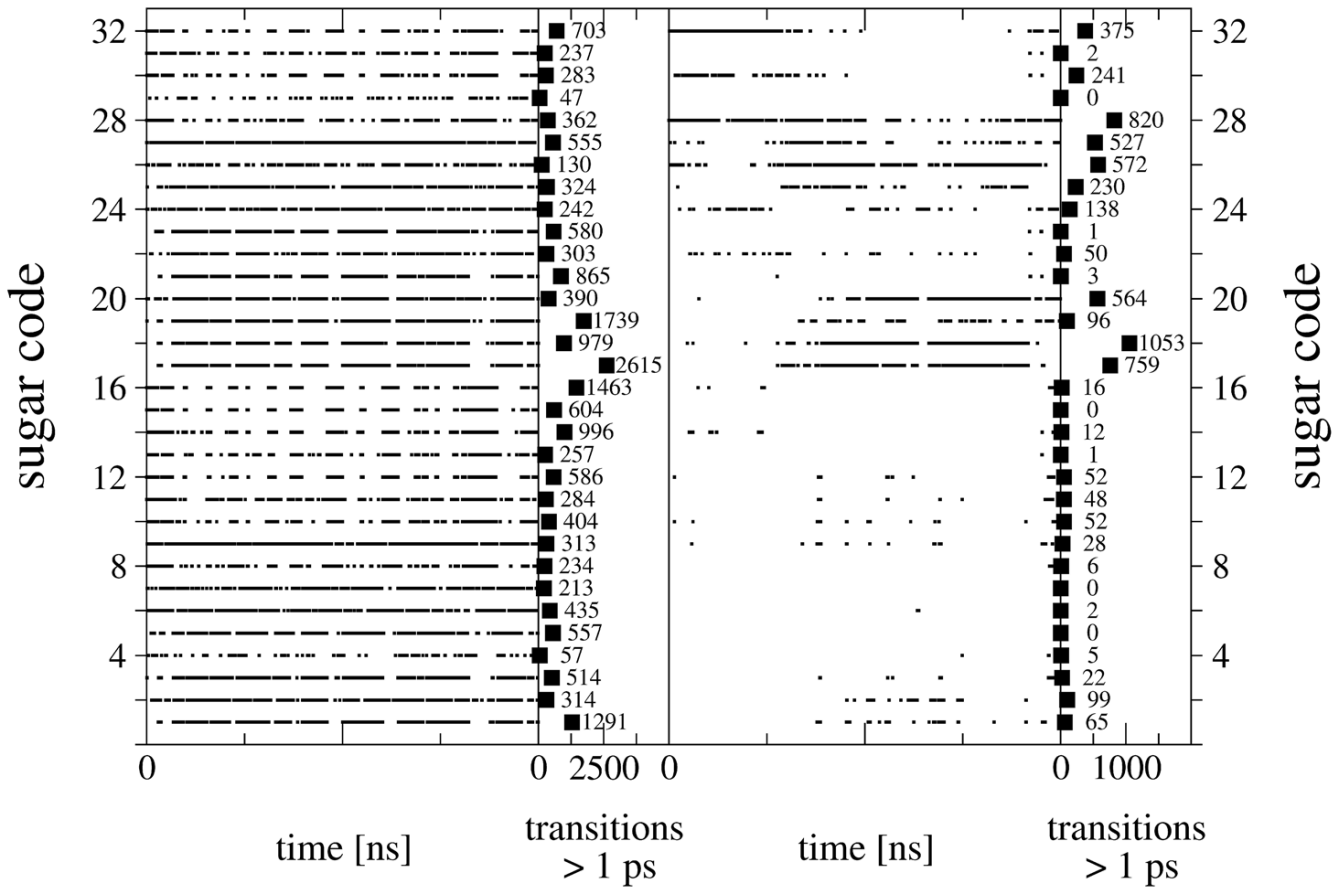
Indicated are (i) the occurrences of the 32 stereoisomers as a function of time and (ii) the number of occurrences with a lifetime ≥1 ps for a particular stereoisomer. The left two panels are derived from the 100 ns MD simulation of system 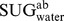, the right two panels represent the 50 ns MD simulation of system 
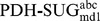
 (pose A), which was selected as a representative example for the PDH-SUG complex.

Fig. 4 shows (i) the occurrence of each of the 32 stereoisomers as a function of time and (ii) the number of occurrences with a lifetime ≥1 ps. The left two panels are derived from the 100 ns MD simulation of system 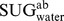, the right two panels represent the 50 ns MD simulation of system 
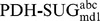
 (pose A), which was selected as a representative example. System 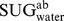 nicely samples all stereoisomers and indicates many transitions between the monosaccharides, leading to good statistics for subsequent analysis. System 
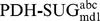
 (pose A) shows significantly less sampling and transitions of the stereoisomers. Therefore, six MD simulations (systems 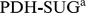, 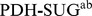, 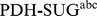; two independent runs each) were conducted for each pose as mentioned previously. In some of the simulations of the PDH-SUG complexes, the unphysical reference state compound was observed to leave the active site. This may very well represent the proper behavior of these molecules, but unbound mixtures of PDH and SUG are (i) not expected to be relevant for real molecules binding to PDH and (ii) not part of the thermodynamic cycle to calculate the binding free energies according to equations (4) and (5). For this reason, simulations 
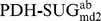
 and 
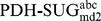
 for pose A and 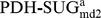 and 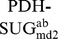 for pose B were excluded from the following analyses and four independent simulations of each pose remained. For the time series of relevant distances between PDH and SUG for all simulations see Figures S1 and S2 in the supplementary material. The remaining four MD simulations for each pose were exponentially averaged according to equation (9) to calculate the free-energy differences (ΔG) of individual stereoisomers and their relative binding free energies (ΔΔG_bind_). According to Fig. 4, system 
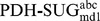
 (pose A) clearly samples l-stereoisomers (sugar code 17–32; 5^th^ digit of improper dihedral code is 4) better than d-stereoisomers (sugar code 1–16; 5^th^ digit of improper dihedral code is 2). This is not surprising, as the transitions of the large CH_2_-OH group attached at this position are sterically the most hindered (see Fig. 2).

**Figure 5:**
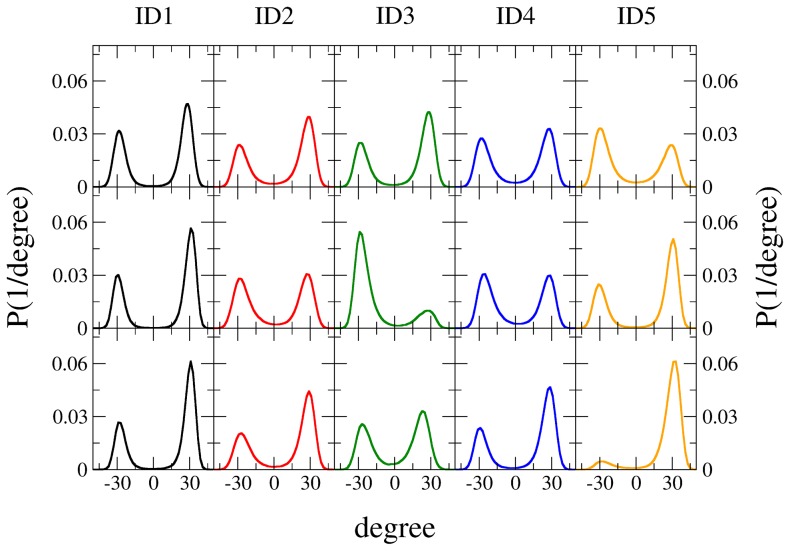
Distributions of all five improper dihedrals (ID) for the MD simulations in water and in protein. For system 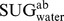 (top five panels), the distributions of the ID are derived from the single 100 ns MD simulation, which sampled all 32 possible stereoisomers (also compare to Fig. 4, left two panels). For system PDH-SUG (pose A) (middle five panels), and for system PDH-SUG (pose B) (lowest five panels), the occurrences as observed in the four selected MD simulations were arithmetically averaged. Coloring scheme according to Fig. 2A.

Fig. 5 shows the distributions of all five improper dihedrals (ID) for the MD simulations in water and in protein. For system 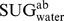 (top five panels in Fig. 5), the distributions of the ID are derived from the single 100 ns MD simulation, which nicely sampled all 32 possible stereoisomers (see Fig. 4, left two panels). For system PDH-SUG (pose A) (middle five panels in Fig. 5), and for system PDH-SUG (pose B) (lowest five panels in Fig. 5), the occurrences of the IDs of the four selected MD simulations were arithmetically averaged. Except for ID5 in pose B and to a lesser extent ID3 in pose A, all improper dihedrals show fairly equal distributions and consequently very good sampling. In spite of lower occurrences for one configuration, ID5 (pose B) and ID3 (pose A) sample both stereoconfigurations. As mentioned previously, a large CH_2_-OH group is attached at ID5 (compare Fig. 2) and consequently transitions of this group are most sterically hindered in the MD simulations within the protein.

Table 3 lists the free-energy differences (ΔG) of the 32 stereoisomers simulated in system PDH-SUG (pose A or pose B). The reported ΔG values were obtained by exponentially averaging the four selected MD simulations for pose A or pose B. Because of the chiral environment within the protein, the span of ΔG values significantly increased (18.9–101.8 kJ/mol for pose A; 21.7–52.8 kJ/mol for pose B) compared to the MD simulation of system 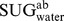 (26.9–45.6 kJ/mol; see Table 1). Moreover, the chiral protein-environment causes significant differences in ΔG between enantiomers. Enantiomers correspond to sugar-pairs with codes 1 and 32, 2 and 31, 3 and 30, etc. The ΔG between enantiomers range from 0.7–60.1 kJ/mol for pose A and from 1.8–24.1 kJ/mol for pose B (in absolute values). In addition, the relative binding free energies (ΔΔG_bind_) are listed in Table 3. They were calculated by subtracting the ΔG values for a certain monosaccharide in the MD simulation of system 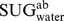 (see Table 1) from the ΔG of the identical monosaccharide in system PDH-SUG in either pose A or pose B (Table 3). Note that these values are relative to the reference states and only differences between them have physical relevance. The range for ΔΔG_bind_ for pose A is −11.7 to 56.2 kJ/mol and −10.1 to 23.1 kJ/mol for pose B. Interestingly, the ΔΔG_bind_ values for the α- and β-anomers of the d-stereoisomers of glucose are among the lowest of all simulated monosaccharides in both poses (range between −10 to −2.9 kJ/mol).

**Table 3:** Free-energy differences (ΔG) of the 32 stereoisomers simulated in system PDH-SUG, with SUG bound to PDH according to pose A or pose B.

Sugar code	Improper dihedral code	Sugar name	Pose A		Pose B	
			ΔG [kJ/mol]	ΔΔG_bind_ [kJ/mol]	ΔG [kJ/mol]	ΔG_bind_ [kJ/mol]
1	22222	β-d-glucose	22.5±1.1	−9.1±1.5	21.7±0.5	−10.0±1.1
2	24222	β-d-mannose	46.7±2.1	8.7±2.3	40.5±2.1	2.5±2.4
3	22242	β-d-galactose	29.2±2.2	−5.3±2.6	28.7±1.1	−5.8±1.7
4	24242	β-d-talose	41.7±2.3	0.8±3.4	39.9±1.2	−1.0±2.8
5	42222	α-d-glucose	31.5±2.3	−2.9±2.6	24.9±1.5	−9.5±1.8
6	44222	α-d-mannose	35.9±1.3	2.1±1.4	28.9±2.3	−4.9±2.4
7	42242	α-d-galactose	33.3±1.9	−2.9±2.3	28.7±1.8	−7.5±2.2
8	44242	α-d-talose	34.5±1.4	−3.3±1.5	30.9±2.1	−6.9±2.2
9	22422	β-d-allose	18.9±0.6	−8.0±0.7	25.7±1.0	−1.1±1.1
10	24422	β-d-altrose	36.6±1.2	8.0±1.2	27.8±1.6	−0.9±1.7
11	22442	β-d-gulose	26.6±1.0	−1.0±1.1	26.6±0.5	−1.0±0.7
12	24442	β-d-idose	40.0±1.0	7.3±1.1	32.5±0.8	−0.2±0.9
13	42422	α-d-allose	35.8±1.0	4.2±1.1	30.6±1.9	−1.0±1.9
14	44422	α-d-altrose	38.2±1.8	8.6±1.8	35.5±1.9	5.8±1.9
15	42442	α-d-gulose	36.0±0.4	4.7±0.5	35.0±1.3	3.7±1.3
16	44442	α-d-idose	36.3±1.0	3.7±1.1	32.4±1.9	−0.2±2.0
17	22224	β-l-glucose	40.1±2.4	9.3±2.4	39.5±2.4	8.7±2.4
18	24224	β-l-mannose	35.3±0.4	5.2±0.5	29.4±2.7	−0.6±2.7
19	22244	β-l-galactose	44.3±1.8	16.3±1.9	45.3±2.1	17.3±2.1
20	24244	β-l-talose	29.9±1.2	−0.5±1.2	38.9±2.2	8.5±2.3
21	42224	α-l-glucose	62.0±2.5	30.8±2.5	51.1±2.5	20.0±2.5
22	44224	α-l-mannose	39.1±1.8	12.0±1.8	40.4±1.6	13.3±1.7
23	42244	α-l-galactose	60.2±2.5	33.2±2.6	50.1±1.2	23.1±1.2
24	44244	α-l-talose	22.6±1.8	−4.3±1.9	40.0±1.9	13.0±1.9
25	22424	β-l-allose	30.6±1.6	−4.6±1.8	52.5±1.5	17.3±1.7
26	24424	β-l-altrose	24.7±0.7	−9.9±1.1	40.2±2.8	5.7±3.0
27	22444	β-l-gulose	37.2±0.9	4.7±0.9	36.2±1.7	3.8±1.8
28	24444	β-l-idose	24.9±0.6	−8.3±1.0	23.1±1.5	−10.1±1.7
29	42424	α-l-allose	101.8±2.5	56.2±2.9	47.3±2.0	1.7±2.4
30	44424	α-l-altrose	33.8±1.1	−2.4±1.8	52.8±1.7	16.5±2.3
31	42444	α-l-gulose	83.1±1.4	44.5±1.7	42.5±1.9	3.9±2.1
32	44444	α-l-idose	21.8±1.7	−11.7±2.0	31.7±1.1	−1.9±1.5

The reported ΔG were obtained by exponentially averaging the four selected MD simulations for each pose according to equation 9. The relative binding free energies (ΔΔG_bind_) were calculated by subtracting the ΔG in the MD simulation of system 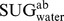 (see Table 1) from the ΔG in system PDH-SUG in the corresponding pose (this table). The improper dihedral code has the same pattern as mentioned in Table 1.

Table 4 gives an overview of the relative binding free energies (ΔΔG_bind_) calculated from the experimentally derived *K*_m_ values [23] and from the combined MD simulations of system PDH-SUG for either pose A or pose B. The experimental values were approximated from the corresponding *K*_m_ values according to the following formula:
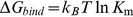
(11)


**Table 4:** Comparison of the relative binding free energies (ΔΔG_bind_) calculated from experiments or MD simulations.

		ΔΔG_bind_ [kJ/mol]		
Sugar name	Michaelis constant *K*_m_ [mM] [23]	Experiment [23]	Simulation	
			Pose A	Pose B
d-glucose	0.82	0.0	0.0	0.0
d-mannose	108	12.3	10.9	5.4
d-galactose	1.05	0.6	3.7	3.4
d-talose	79.1	11.5	5.6	3.6

The experimental values were estimated from the corresponding *K*_m_ values according to the formula Δ*G*  =  *k_B_T* ln *K*_m_. All ΔΔG_bind_ values are reported relative to d-glucose, which was set to zero. Moreover, the ΔΔG_bind_ values for pose A and pose B were not averaged, because the preference of the reference states for a certain pose is unknown.

Experimental data were available only for the four listed d-stereoisomers. Because the α- and β-anomers spontaneously interconvert in solution via mutarotation, they cannot be distinguished in experimental binding. The ΔΔG_bind_ values for pose A or pose B were calculated from the MD simulations by first exponentially averaging the free-energy differences of the α- and β-anomers of the respective d-stereoisomers simulated in system 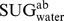 (Table 1) or in system PDH-SUG in pose A or pose B (Table 3). Then, the averaged ΔG values in system 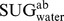 were subtracted from system PDH-SUG (pose A or pose B) to obtain the ΔΔG_bind_. For easier comparison, the ΔΔG_bind_ for d-glucose was set to zero. The ΔΔG_bind_ for pose A and pose B were not averaged, as the preference of the reference states for a certain pose is unknown. The ΔΔG_bind_ values derived from simulations of pose A agree well with experiment. Only the difference for d-talose between the experimental and calculated ΔΔG_bind_ (5.9 kJ/mol) is larger than the thermal noise. The agreement for pose B matches qualitatively, with differences between the experimental and calculated ΔΔG_bind_ values of 6.9 kJ/mol for d-mannose and 7.9 kJ/mol for d-talose, which are both above the thermal noise. To conclude, the ΔΔG_bind_ values derived from simulations and experiments match quite well.

**Figure 6:**
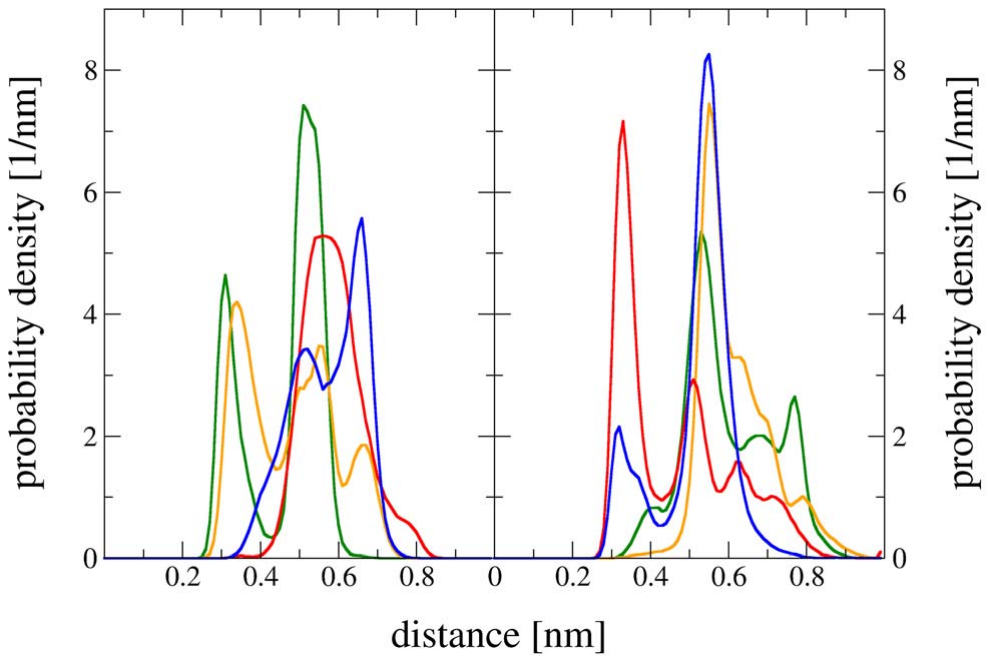
Distances between H-atoms HC1–HC4 of SUG and the N5-atom of FAD. The distances of all four simulations of pose A or pose B of system PDH-SUG were used. Left panel: pose A, right panel: pose B. Color codes match with Fig. 2A: HC1 (green), HC2 (yellow), HC3 (red), and HC4 (blue).

For successful oxidation, a hydride transfer takes place from the SUG-oxidation site to the N5 atom of FAD [13]. Fig. 6 shows the distances between H-atoms HC1–HC4 of SUG and the N5-atom of FAD. The position of the hydrogen atom in our united-atom representation of the reference state was determined according to ideal geometries and a C-H bond length of 0.1 nm. The occurrence of the distances of all four simulations of pose A and pose B were arithmetically averaged. As reported previously [13], a 0.3 nm cutoff was considered in order for a hydride transfer to occur between HC1–HC4 and N5. Color codes are the same as in Fig. 2A. In pose A (left panel), only HC1 (green) and HC2 (yellow) are below the mentioned cutoff. In pose B, only HC3 (red) and HC4 (blue) are below the 0.3 nm cutoff. This corresponds very well to previously published data for d-glucose oxidation by PDH, where pose A represents the C2 oxidation mode and pose B the C3 oxidation mode of this particular sugar [13].

**Figure 7:**
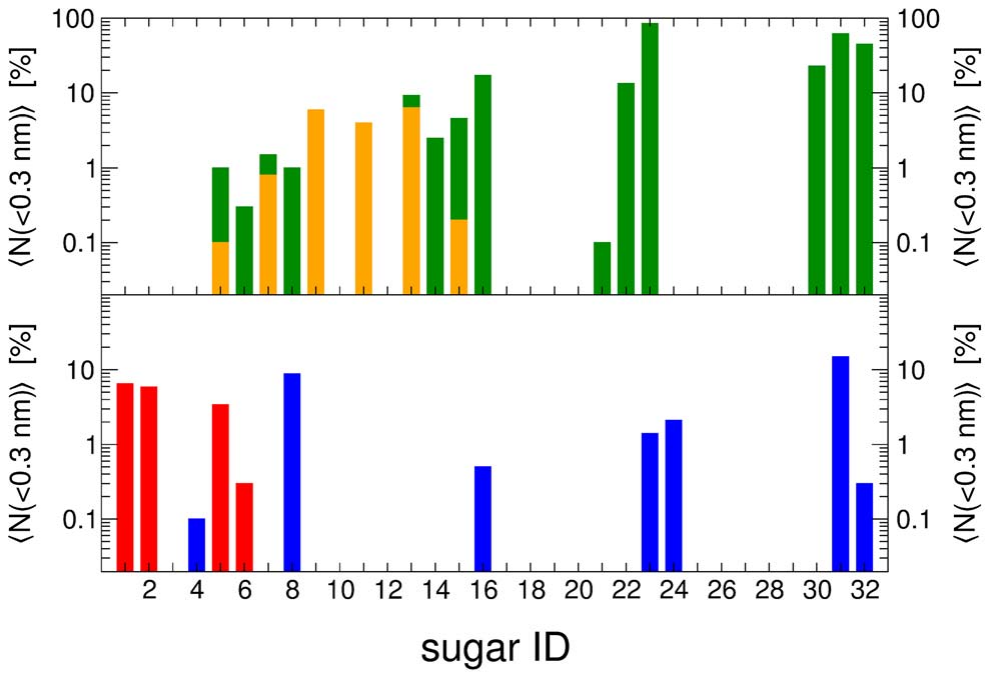
Reactive distances below 0.3 nm between hydrogens attached to C1–C4 of SUG and the N5-atom of FAD for pose A (upper panel) and pose B (lower panel). Color codes match with Fig. 2A: HC1 (green), HC2 (yellow), HC3 (red), and HC4 (blue). The bars consisting of more than one color are non-additive.

In Fig. 7, the average number of observations of distances between hydrogens attached to C1–C4 and the N5-atom in FAD below 0.3 nm for pose A and pose B are shown as calculated for all monosaccharides using equations 7 and 6. The bars in this logarithmic diagram are non-additive. These reactive distances are compared to the experimentally detected oxidation products. When the distances are below 0.3 nm, we will use the ΔΔG_bind_ to evaluate the likelihood of the corresponding monosaccharide to bind to PDH and consequently for a reaction to take place. Note that also low values of 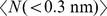 can already explain reactions, as substrate binding can easily be a much slower process than the actual reaction. The ΔΔG_bind_ value of β-d-glucose is set to zero in each pose and the ΔΔG_bind_ values of the other sugars are reported here relative to β-d-glucose. Experimental d-glucose conversions yield (di-)oxidations at C2 and C3 [25]. This observation is reproduced by MD simulations: in pose A, 0.1% of the HC2-N5 distance is below 0.3 nm in β-d-glucose; in pose B, the HC3-N5 distance is below the chosen cutoff for 3.4% (α-d-glucose) and 6.5% (β-d-glucose). Again, this observation corresponds very well to our previously published work [13], where d-glucose is oxidized at C2 in pose A and at C3 in pose B. Experimentally, l-glucose is observed to have C2- and C3 (di-)oxidations as well. However, in our MD simulations we do not see any of the relevant HC2-N5 or HC3-N5 distances below the 0.3 nm cutoff. Moreover, its relative activity was experimentally measured to be 42% of d-glucose [25], which does not correspond to the predicted highly unfavorable ΔΔG_bind_ values between 18.5–39.9 kJ/mol for l-glucose bound in either pose. For d-galactose, MD simulations gave a HC2-N5 distance below 0.3 nm 0.8% of the time for α-d-galactose in pose A, which corresponds to its experimentally observed C2 oxidation [25]. Moreover, we predict a relatively favorable ΔΔG_bind_ value of 6.2 kJ/mol for α-d-galactose in pose A. The sugar d-mannose is a substrate for PDH, however, its oxidation sites have not yet been determined experimentally. The most prominent reactive distance for this sugar is HC3-N5 (β-d-mannose bound in pose B), which is below 0.3 nm for 5.9% of the time and has a predicted ΔΔG_bind_ value of 12.5 kJ/mol. For d-allose, C1 oxidation has been experimentally reported [25]. In the MD simulations, the corresponding HC1-N5 distance is below the 0.3 nm cutoff 2.9% of the time for α-d-allose (pose A). The predicted ΔΔG_bind_ value for α-d-allose (pose A) is 13.3 kJ/mol, corresponding to the experimentally determined relative activity of 15% of d-glucose [25]. Experiments revealed solely C1 oxidation for d-talose, which was reproduced by MD simulations with the HC1-N5 distance below 0.3 nm 1.0% of the time for α-d-talose bound to PDH in pose A. The predicted ΔΔG_bind_ value for α-d-talose (pose A) is 5.8 kJ/mol, which agrees qualitatively and to a lesser extent quantitatively with the experimentally determined binding affinity according to its *K*_m_ value (see also Table 4). The HC4-N5 distance for α-d-talose (pose B) is below the 0.3 nm cutoff 8.8% of the time and the corresponding ΔΔG_bind_ value is reasonably low for binding (3.1 kJ/mol). Nevertheless, C4 oxidation is not reported experimentally, which might be caused by steric clashes of the adjacent hydroxymethyl-group attached to the C5 carbon resulting in poor binding. The last experimentally determined oxidation site for a monosaccharide investigated in this study is available for d-gulose, which is oxidized at C1 [25] only. In the MD simulations, the HC1-N5 distance for α-d-gulose (pose A) is indeed below 0.3 nm 4.4% of the time. The activity of d-gulose was reported to be 7% of d-glucose [25], which corresponds to an unfavorable ΔΔG_bind_ value for α-d-gulose (pose A) of 13.9 kJ/mol.

In addition to the experimentally determined oxidation sites, we made some striking observations during our distance analyses, which can direct future experiments. High percentages of reactive poses suggesting C1 oxidation are observed for the following sugars bound in pose A: α-d-idose (17.2%), α-l-mannose (13.3%), α-l-galactose (85.5%), α-l-altrose (22.9%), α-l-gulose (62.3%), and α-l-idose (45.0%). Some of these possible oxidation products can be neglected, as the predicted ΔΔG_bind_ value for the corresponding monosaccharides is very unfavorable in pose A: α-l-gulose (53.6 kJ/mol), α-l-galactose (42.3 kJ/mol), and α-l-mannose (21.1 kJ/mol). Others could have low, but measurable activities: α-d-idose (12.8 kJ/mol) and α-l-altrose (6.7 kJ/mol). Lastly, oxidation for α-l-idose (−2.6 kJ/mol) with HC1-N5 below 0.3 nm 45% of the time is predicted in pose A. For pose B, we predict HC4 oxidation of α-l-gulose, for which the HC4-N5 distance is below 0.3 nm 14.9% of the time and the predicted ΔΔG_bind_ value is 13.9 kJ/mol, allowing for low but measurable activity.

Interestingly, monosaccharides bound to PDH in pose A, for which additional oxidations were observed, are all α-compounds and oxidized at HC1. This can be rationalized, as the hydroxyl-group attached to C1 defines whether a sugar is an α- or β-anomer. Consequently, if the HC1-N5 is within the reactive distance, the hydroxyl-group attached to that C1 has to be on the opposite side of the HC1, which (in pose A) corresponds to the α-anomer of the respective sugars.

## Conclusions

In this study, we presented a generalized approach to simulate monosaccharide solvation in water, as well as binding and product formation in the enzyme PDH. Introducing changes to the monosaccharide topology according to Fig. 2 created systems SUG^a^, SUG^ab^, and SUG^abc^, out of which system SUG^ab^ was selected as the most suitable reference state for subsequent analysis in water. This allowed for sampling of all 32 possible aldohexopyranoses in only one MD simulation of the reference compound in water or using a handful of simulations of the reference state compounds within PDH.

Free energy calculations according to the one-step perturbation method revealed that systems 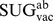 and 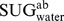 show a similar range of relative free energies for the simulated monosaccharides. Moreover, the relative free energies for the enantiomer-pairs (sugar codes 1 and 32, 2 and 31, etc.) match very well within systems 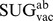 and 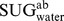. Because both vacuum and water represent an achiral environment, this outcome is expected and gives confidence in the conducted simulations. We reported calculated values for the relative solvation free energies (ΔΔG_solv_) of all 32 aldohexopyranoses (Table 1). To the best of our knowledge, these ΔΔG_solv_ values have not been reported previously, giving new fundamental insights into the solvation of aldohexopyranoses. For all simulated pyranose d-stereoisomers, we report the free energy differences of the corresponding β/α-anomers in water (ΔG_β−α_). The deviations from experimental values [23], [25] are very small (within a range of 0.0–2.3 kJ/mol), further increasing the confidence in the conducted simulations. In addition, the pyranose ring conformations for each of the 32 stereoisomers were investigated. Sugars with codes 1–16 (d-stereoisomers) occurred predominantly in the ^4^C_1_ chair conformation and sugars with codes 17–32 (l-stereoisomers) in the ^1^C_4_ chair conformation. These results are in line with the experimentally obtained preferences of ring conformations for d- or l-stereoisomers [55]–[57], furthermore strengthening the validity of the performed simulations.

For system PDH-SUG, six MD simulations of 50 ns each were performed for either pose A or pose B. Of these six, two were discarded for each pose from subsequent analysis, because the monosaccharide left the active site. For each pose, the results of the four selected MD simulations were exponentially averaged. All 32 possible stereoisomers were sampled extensively in pose A and pose B. Compared to system 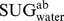 (26.9–45.6 kJ/mol), the chiral protein environment caused a significant increase in the span of the free-energy differences (ΔG): 18.9–101.8 kJ/mol for pose A and 21.7–52.8 kJ/mol for pose B. The relative binding free energies (ΔΔG_bind_) range between −11.7 to 56.2 kJ/mol for pose A and between -10.1 to 23.1 kJ/mol for pose B. Where available, the calculated ΔΔG_bind_ values were compared to the experimental binding free energies estimated from *K*_m_ values. For pose A, the agreement is quite reasonable. Pose B shows qualitative agreement between calculated and experimental ΔΔG_bind_ values. Taking an arbitrary cutoff of +15 kJ/mol relative to β-d-glucose for binding, the predicted binding affinities indicate that measurable binding free energies can be expected for 21 monosaccharides in pose A as well as in pose B. This is in line with the expected promiscuity of the PDH enzyme and suggests that these monosaccharides can be anticipated to inhibit the enzyme and possibly are also substrates themselves.

A distance analysis between hydrogens attached to the sugar carbons 1–4 and the N5 atom of FAD revealed that monosaccharide oxidation is possible at HC1 or HC2 in pose A and at HC3 or HC4 in pose B, which is in line with previously published findings [13]. We could reproduce the experimentally detected oxidation products by monitoring the HCX–N5 distance for d-glucose, d-galactose, d-allose, d-talose, and d-gulose. Only for l-glucose, the experimentally observed C2- or C3- oxidation could not be reproduced by the HCX-N5 distance analysis. With a combination of HCX-N5 distance analysis and binding free energy calculations, we predict oxidation products for some sugars, which have not yet been reported experimentally: low but measurable oxidation at HC1 for l-altrose and d-idose as well as at HC3 for d-mannose and at HC4 for l-gulose; strong oxidation at HC1 for l-idose – a challenge for future experiments.

To conclude, this study presents a generalized approach to simulate all 32 possible aldohexopyranoses in the course of just a few simulations. It contributes to the rationalization of PDH's substrate promiscuity with a combination of binding free energies and distance analyses for each sugar. This provides insights into PDH's applicability in bioelectrochemistry. We believe that this approach is readily transferable to other promiscuous enzymes, whose substrates differ mainly in the stereochemistry of their reactive groups.

## Supporting Information

Figure S1Distances between selected atoms of PDH and SUG in system PDH-SUG (pose A) used to monitor whether SUG left the binding site. In the upper two panels, the SUG-topology was altered according to SUG^a^, in the middle two panels according to SUG^ab^, and in the lowest two panels according to SUG^abc^ (see Fig. 2 and text for more details). The first column represents the first repeat of the MD simulations for each of the changed SUG-topologies (md1) and the second column the second repeat (md2). Colors indicate the following distances: Glu-392(NE2)–SUG(C4) (black), Val-511(C)–SUG(C1) (red), and Val-511(N)–SUG(C5) (green). For pose A, the MD simulations of systems 
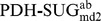
 and 
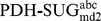
 were discarded, because the SUG left PDH's active site.(TIFF)

Figure S2Distances between selected atoms of PDH and SUG in system PDH-SUG (pose B) used to monitor whether SUG left the binding site. In the upper two panels, the SUG-topology was altered according to SUG^a^, in the middle two panels according to SUG^ab^, and in the lowest two panels according to SUG^abc^ (see Fig. 2 and text for more details). The first column represents the first repeat of the MD simulations for each of the changed SUG-topologies (md1) and the second column the second repeat (md2). Colors indicate the following distances: Val-511(C)–SUG(C3) (black), Gln-392(CD)–SUG(C1) (red), and Val-511(N)–SUG(C4) (green). For pose B, the MD simulations of systems 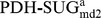 and 
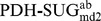
 were discarded, because the SUG left PDH's active site. Although SUG left the active site in system 
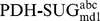
 in the last 2–3 ns, it was still used for subsequent analysis.(TIFF)

Table S1 Topology of system β-d-glucose in the GROMOS 53A6 force field. Changes made to the topology to obtain the reference states are written in the corresponding line after the hash-symbol in bold and are in agreement with Fig. 2. The improper dihedral (ID) type code listed in the IMPDIHEDRAL-block corresponds to the ID code in Tables 1 and 3, where the ID-sequence has been changed from the original SUG-topology according to Fig. 2A.
(DOCX)
