# Supplementary figures and images for: Pyranose Dehydrogenase Ligand Promiscuity: A Generalized Approach to Simulate Monosaccharide Solvation, Binding, and Product Formation
Source: PLoS Comput Biol. 2014 Dec 11;10(12):e1003995. doi: 10.1371/journal.pcbi.1003995 (PMC4263366; doi:10.1371/journal.pcbi.1003995)

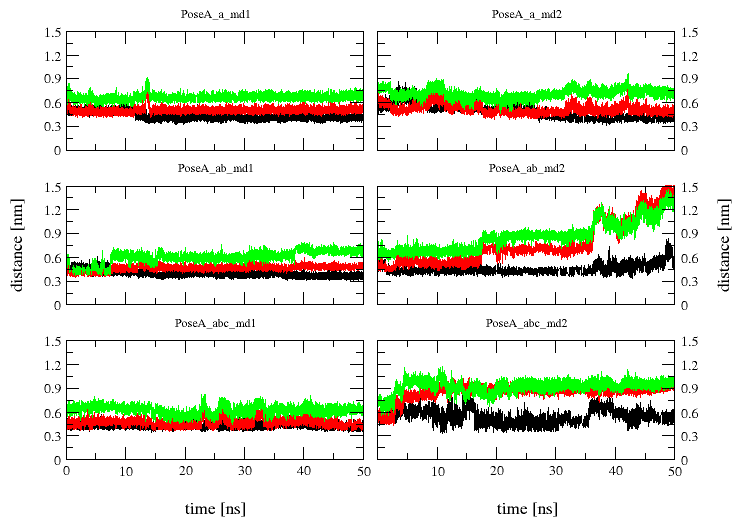

Supplement: Figure S1 — Distances between selected atoms of PDH and SUG in system PDH-SUG (pose A) used to monitor whether SUG left the binding site. In the upper two panels, the SUG-topology was altered according to SUGa, in the middle two panels according to SUGab, and in the lowest two panels according to SUGabc (see Fig. 2 and text for more details). The first column represents the first repeat of the MD simulations for each of the changed SUG-topologies (md1) and the second column the second repeat (md2). Colors indicate the following distances: Glu-392(NE2)–SUG(C4) (black), Val-511(C)–SUG(C1) (red), and Val-511(N)–SUG(C5) (green). For pose A, the MD simulations of systems and were discarded, because the SUG left PDH's active site. (TIFF) [file pcbi.1003995.s001.tiff]

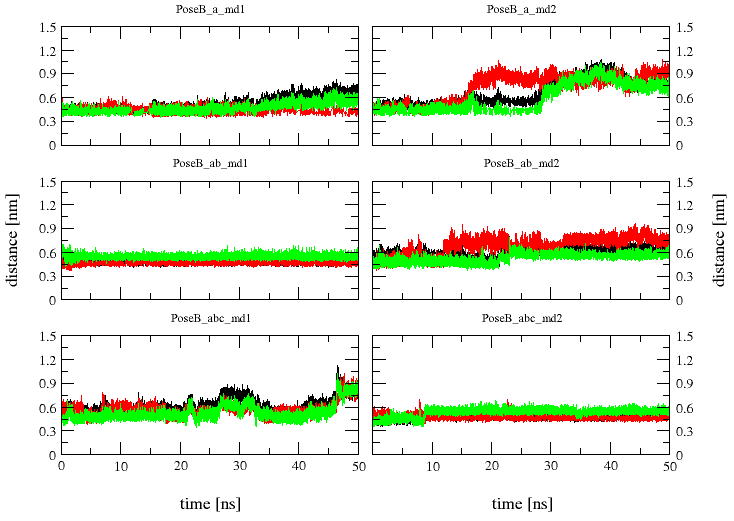

Supplement: Figure S2 — Distances between selected atoms of PDH and SUG in system PDH-SUG (pose B) used to monitor whether SUG left the binding site. In the upper two panels, the SUG-topology was altered according to SUGa, in the middle two panels according to SUGab, and in the lowest two panels according to SUGabc (see Fig. 2 and text for more details). The first column represents the first repeat of the MD simulations for each of the changed SUG-topologies (md1) and the second column the second repeat (md2). Colors indicate the following distances: Val-511(C)–SUG(C3) (black), Gln-392(CD)–SUG(C1) (red), and Val-511(N)–SUG(C4) (green). For pose B, the MD simulations of systems and were discarded, because the SUG left PDH's active site. Although SUG left the active site in system in the last 2–3 ns, it was still used for subsequent analysis. (TIFF) [file pcbi.1003995.s002.tiff]
