# Supplementary material for: Pyranose Dehydrogenase Ligand Promiscuity: A Generalized Approach to Simulate Monosaccharide Solvation, Binding, and Product Formation
Source: PLoS Comput Biol. 2014 Dec 11;10(12):e1003995. doi: 10.1371/journal.pcbi.1003995 (PMC4263366; doi:10.1371/journal.pcbi.1003995)
Supplement: Table S1 — Topology of system β-d-glucose in the GROMOS 53A6 force field. Changes made to the topology to obtain the reference states are written in the corresponding line after the hash-symbol in bold and are in agreement with Fig. 2. The improper dihedral (ID) type code listed in the IMPDIHEDRAL-block corresponds to the ID code in Tables 1 and 3, where the ID-sequence has been changed from the original SUG-topology according to Fig. 2A. (DOCX) [file pcbi.1003995.s003.docx]

Topology of system β-d-glucose in the GROMOS 53A6 force field. Changes made to the topology to obtain the reference states are written in the corresponding line after the hash-symbol ­­­­in bold and are in agreement with Figure 2. The improper dihedral (ID) type code listed in the IMPDIHEDRAL-block corresponds to the ID code in Table 1 and 3, where the ID-sequence has been changed from the original SUG-topology according to Figure 2A.

RESNAME

# NRAA2: number of residues in a solute molecule

1

# AANM: residue names

GB4P

END

SOLUTEATOM

# NRP: number of solute atoms

17

# ATNM: atom number

# MRES: residue number

# PANM: atom name of solute atom

# IAC: integer (van der Waals) atom type code

# MASS: mass of solute atom

# CG: charge of solute atom

# CGC: charge group code (0 or 1)

# INE: number of excluded atoms

# INE14: number of 1-4 interactions

# ATNM MRES PANM IAC MASS CG CGC INE

# INE14

1 1 HO4 21 1.00800 0.41000 0 2 2 3

2 4 13

2 1 O4 3 15.99940 -0.64200 0 3 3 4 13

4 5 7 10 14

3 1 C4 14 13.01900 0.23200 1 6 4 5 7 10 13 14

4 6 8 11 15

4 1 C3 14 13.01900 0.23200 0 6 5 6 7 8 13 15

4 9 10 14 16

5 1 O3 3 15.99940 -0.64200 0 2 6 7

3 8 13 15

6 1 HO3 21 1.00800 0.41000 1 0

1 7

7 1 C2 14 13.01900 0.23200 0 5 8 9 14 15 16

2 13 17

8 1 O2 3 15.99940 -0.64200 0 2 9 15

2 14 16

9 1 HO2 21 1.00800 0.41000 1 0

1 15

10 1 C6 15 14.02700 0.23200 0 4 11 12 13 14

1 15

11 1 O6 3 15.99940 -0.64200 0 2 12 13

1 14

12 1 HO6 21 1.00800 0.41000 1 0

1 13

13 1 C5 14 13.01900 0.37600 0 2 14 15

1 16

14 1 O5 3 15.99940 -0.48000 0 3 15 16 17

0

15 1 C1 14 13.01900 0.23200 0 2 16 17

0

16 1 O1 3 15.99940 -0.53800 0 1 17

0

17 1 HO1 21 1.00800 0.41000 1 0

0

END

BONDSTRETCHTYPE

# NBTY: number of covalent bond types

52

# CB: quartic force constant

# CHB: harmonic force constant

# B0: bond length at minimum energy

# CB CHB B0

1.57000e+07 3.14000e+05 1.00000e-01

1.87000e+07 3.74000e+05 1.00000e-01

1.23000e+07 2.92273e+05 1.09000e-01

3.70000e+07 9.28256e+05 1.12000e-01

1.66000e+07 5.02283e+05 1.23000e-01

1.34000e+07 4.18750e+05 1.25000e-01

1.20000e+07 4.18176e+05 1.32000e-01

8.87000e+06 3.13803e+05 1.33000e-01

1.06000e+07 3.75007e+05 1.33000e-01

1.18000e+07 4.17460e+05 1.33000e-01

# 10

1.05000e+07 3.77076e+05 1.34000e-01

1.17000e+07 4.20170e+05 1.34000e-01

1.02000e+07 3.77318e+05 1.36000e-01

1.10000e+07 4.18968e+05 1.38000e-01

8.66000e+06 3.34640e+05 1.39000e-01

1.08000e+07 4.17334e+05 1.39000e-01

8.54000e+06 3.34768e+05 1.40000e-01

8.18000e+06 3.34546e+05 1.43000e-01

9.21000e+06 3.76671e+05 1.43000e-01

6.10000e+06 2.51225e+05 1.43500e-01

# 20

8.71000e+06 3.76429e+05 1.47000e-01

5.73000e+06 2.51020e+05 1.48000e-01

7.64000e+06 3.34693e+05 1.48000e-01

8.60000e+06 3.76749e+05 1.48000e-01

8.37000e+06 3.76650e+05 1.50000e-01

5.43000e+06 2.50909e+05 1.52000e-01

7.15000e+06 3.34749e+05 1.53000e-01

4.84000e+06 2.50915e+05 1.61000e-01

4.72000e+06 2.50811e+05 1.63000e-01

2.72000e+06 1.72361e+05 1.78000e-01

# 30

5.94000e+06 3.76406e+05 1.78000e-01

5.62000e+06 3.76416e+05 1.83000e-01

3.59000e+06 2.51077e+05 1.87000e-01

6.40000e+05 5.01811e+04 1.98000e-01

6.28000e+05 5.02400e+04 2.00000e-01

5.03000e+06 4.18657e+05 2.04000e-01

5.40000e+05 5.27483e+04 2.21000e-01

2.32000e+07 4.64000e+05 1.00000e-01

1.21000e+07 2.92820e+05 1.10000e-01

8.12000e+06 5.01908e+05 1.75800e-01

# 40

8.04000e+06 3.76417e+05 1.53000e-01

4.95000e+06 3.71825e+05 1.93799e-01

8.10000e+06 5.01811e+05 1.76000e-01

1.31000e+07 4.19259e+05 1.26500e-01

1.03000e+07 3.75435e+05 1.35000e-01

8.71000e+06 4.64532e+05 1.63299e-01

2.68000e+06 2.93088e+05 2.33839e-01

2.98000e+06 5.02215e+05 2.90283e-01

2.39000e+06 3.73116e+05 2.79388e-01

2.19000e+06 3.71385e+05 2.91189e-01

# 50

3.97000e+06 3.42526e+05 2.07700e-01

3.04000e+06 5.02225e+05 2.87407e-01

END

BONDH

# NBONH: number of bonds involving H atoms in solute

5

# IBH, JBH: atom sequence numbers of atoms forming a bond

# ICBH: bond type code

# IBH JBH ICBH

1 2 1

5 6 1

8 9 1

11 12 1

16 17 1

END

BOND

# NBON: number of bonds NOT involving H atoms in solute

12

# IB, JB: atom sequence numbers of atoms forming a bond

# ICB: bond type code

# IB JB ICB

2 3 20

3 4 26

3 13 26

4 5 20

4 7 26

7 8 20

7 15 26

10 11 20

10 13 26

13 14 20

# 10

14 15 20

15 16 20

END

BONDANGLEBENDTYPE

# NTTY: number of bond angle types

54

# CT: force constant (based on potential

# harmonic in the angle cosine)

# CHT: force constant (based on potential

# harmonic in the angle)

# T0: bond angle at minimum energy in degrees

# CT CHT T0

3.80000e+02 1.15501e-01 9.00000e+01

4.20000e+02 1.27686e-01 9.00000e+01

4.05000e+02 1.21771e-01 9.60000e+01

4.75000e+02 1.40083e-01 1.00000e+02

4.20000e+02 1.21222e-01 1.03000e+02

4.90000e+02 1.40285e-01 1.04000e+02

4.65000e+02 1.27888e-01 1.08000e+02

2.85000e+02 7.69125e-02 1.09500e+02

3.20000e+02 8.63861e-02 1.09500e+02

3.80000e+02 1.02627e-01 1.09500e+02

# 10

4.25000e+02 1.14807e-01 1.09500e+02

4.50000e+02 1.21574e-01 1.09500e+02

5.20000e+02 1.40521e-01 1.09500e+02

4.50000e+02 1.21423e-01 1.09600e+02

5.30000e+02 1.40487e-01 1.11000e+02

5.45000e+02 1.40451e-01 1.13000e+02

5.00000e+01 1.22966e-02 1.15000e+02

4.60000e+02 1.14885e-01 1.15000e+02

6.10000e+02 1.52417e-01 1.15000e+02

4.65000e+02 1.14219e-01 1.16000e+02

# 20

6.20000e+02 1.52361e-01 1.16000e+02

6.35000e+02 1.53360e-01 1.17000e+02

3.90000e+02 8.89104e-02 1.20000e+02

4.45000e+02 1.01476e-01 1.20000e+02

5.05000e+02 1.15184e-01 1.20000e+02

5.30000e+02 1.20895e-01 1.20000e+02

5.60000e+02 1.27749e-01 1.20000e+02

6.70000e+02 1.52880e-01 1.20000e+02

7.80000e+02 1.78011e-01 1.20000e+02

6.85000e+02 1.53127e-01 1.21000e+02

# 30

7.00000e+02 1.53174e-01 1.22000e+02

4.15000e+02 8.87438e-02 1.23000e+02

7.30000e+02 1.52669e-01 1.24000e+02

3.75000e+02 7.64902e-02 1.25000e+02

7.50000e+02 1.53141e-01 1.25000e+02

5.75000e+02 1.14487e-01 1.26000e+02

6.40000e+02 1.27447e-01 1.26000e+02

7.70000e+02 1.53365e-01 1.26000e+02

7.60000e+02 1.27755e-01 1.32000e+02

2.21500e+03 1.21127e-01 1.55000e+02

# 40

9.13500e+04 7.26402e-02 1.80000e+02

4.34000e+02 1.17243e-01 1.09500e+02

4.84000e+02 1.33765e-01 1.07570e+02

6.32000e+02 1.66891e-01 1.11300e+02

4.69000e+02 1.40245e-01 9.74000e+01

5.03000e+02 1.40260e-01 1.06750e+02

4.43000e+02 1.21084e-01 1.08530e+02

6.18000e+02 1.67047e-01 1.09500e+02

5.07000e+02 1.40086e-01 1.07600e+02

4.48000e+02 1.21033e-01 1.09500e+02

# 50

5.24000e+02 1.40180e-01 1.10300e+02

5.32000e+02 1.40257e-01 1.11400e+02

6.36000e+02 1.53054e-01 1.17200e+02

6.90000e+02 1.52948e-01 1.21400e+02

END

BONDANGLEH

# NTHEH: number of bond angles involving H atoms in solute

5

# ITH, JTH, KTH: atom sequence numbers

# of atoms forming a bond angle in solute

# ICTH: bond angle type code

# ITH JTH KTH ICTH

1 2 3 12

4 5 6 12

7 8 9 12

10 11 12 12

15 16 17 12

END

BONDANGLE **#Modified according to Figure 2C**

# NTHE: number of bond angles NOT

# involving H atoms in solute

17

# IT, JT, KT: atom sequence numbers of atoms

# forming a bond angle

# ICT: bond angle type code

# IT JT KT ICT

2 3 4 9 **#Last value set to 8**

2 3 13 9 **#Last value set to 8**

4 3 13 8

3 4 5 9 **#Last value set to 8**

3 4 7 8

5 4 7 9 **#Last value set to 8**

4 7 8 9 **#Last value set to 8**

4 7 15 8

8 7 15 9 **#Last value set to 8**

11 10 13 9

# 10

3 13 10 8

3 13 14 9 **#Last value set to 8**

10 13 14 9 **#Last value set to 8**

13 14 15 10 **#Last value set to 8**

7 15 14 9 **#Last value set to 8**

7 15 16 9 **#Last value set to 8**

14 15 16 9 **#Last value set to 8**

END

IMPDIHEDRALTYPE

# NQTY: number of improper dihedrals

4

# CQ: force constant of improper dihedral per degrees square

# Q0: improper dihedral angle at minimum energy in degrees

# CQ Q0

5.10000e-02 0.00000e+00

1.02000e-01 3.52644e+01

2.04000e-01 0.00000e+00

1.02000e-01 -3.52644e+01

END

IMPDIHEDRALH

# NQHIH: number of improper dihedrals

# involving H atoms in the solute

0

# IQH,JQH,KQH,LQH: atom sequence numbers

# of atoms forming an improper dihedral

# ICQH: improper dihedral type code

# IQH JQH KQH LQH ICQH

END

IMPDIHEDRAL **#Modified according to Figure 2A**

# NQHI: number of improper dihedrals NOT

# involving H atoms in solute

5 **#This value was set to 0**

# IQ,JQ,KQ,LQ: atom sequence numbers of atoms

# forming an improper dihedral

# ICQ: improper dihedral type code

# IQ JQ KQ LQ ICQ

3 10 14 13 2 **#This line was deleted**

4 5 7 3 2 **#This line was deleted**

7 14 16 15 2 **#This line was deleted**

13 2 4 3 2 **#This line was deleted**

15 4 8 7 2 **#This line was deleted**

END

TORSDIHEDRALTYPE

# NPTY: number of dihedral types

41

# CP: force constant

# PD: phase-shift angle

# NP: multiplicity

# CP PD NP

2.67000 180.00000 1

3.41000 180.00000 1

4.97000 180.00000 1

5.86000 180.00000 1

9.35000 180.00000 1

9.45000 180.00000 1

2.79000 0.00000 1

5.35000 0.00000 1

1.53000 180.00000 2

5.86000 180.00000 2

# 10

7.11000 180.00000 2

16.70000 180.00000 2

24.00000 180.00000 2

33.50000 180.00000 2

41.80000 180.00000 2

0.00000 0.00000 2

0.41800 0.00000 2

2.09000 0.00000 2

3.14000 0.00000 2

5.09000 0.00000 2

# 20

16.70000 0.00000 2

1.05000 0.00000 3

1.26000 0.00000 3

1.30000 0.00000 3

2.53000 0.00000 3

2.93000 0.00000 3

3.19000 0.00000 3

3.65000 0.00000 3

3.77000 0.00000 3

3.90000 0.00000 3

# 30

4.18000 0.00000 3

4.69000 0.00000 3

5.44000 0.00000 3

5.92000 0.00000 3

7.69000 0.00000 3

8.62000 0.00000 3

9.50000 0.00000 3

0.00000 0.00000 4

1.00000 180.00000 6

1.00000 0.00000 6

# 40

3.77000 0.00000 6

END

DIHEDRALH

# NPHIH: number of dihedrals involving H atoms in solute

6

# IPH, JPH, KPH, LPH: atom sequence numbers

# of atoms forming a dihedral

# ICPH: dihedral type code

# IPH JPH KPH LPH ICPH

1 2 3 4 30

7 4 5 6 30

15 7 8 9 30

13 10 11 12 30

14 15 16 17 2

14 15 16 17 32

END

DIHEDRAL **#Modified according to Figure 2B**

# NPHI: number of dihedrals NOT involving H atoms in solute

19

# IP, JP, KP, LP: atom sequence numbers

# of atoms forming a dihedral

# ICP: dihedral type code

# IP JP KP LP ICP

2 3 4 5 18 **#Last value set to 17**

2 3 4 7 17

13 3 4 5 17

13 3 4 7 34 **#Last value set to 22**

2 3 13 10 17

4 3 13 14 17

4 3 13 14 34 **#Last value set to 22**

3 4 7 8 17

3 4 7 15 34 **#Last value set to 22**

5 4 7 8 18 **#Last value set to 17**

# 10

5 4 7 15 17

4 7 15 14 17

4 7 15 14 34 **#Last value set to 22**

4 7 15 16 17

8 7 15 16 18 **#Last value set to 17**

11 10 13 14 5

11 10 13 14 37

3 13 14 15 29 **#Last value set to 22**

13 14 15 7 29 **#Last value set to 22**

END

CROSSDIHEDRALH

# NPHIH: number of cross dihedrals involving H atoms in solute

0

# APH, BPH, CPH, DPH, EPH, FPH, GPH, HPH: atom sequence numbers

# of atoms forming a dihedral

# ICCH: dihedral type code

# APH BPH CPH DPH EPH FPH GPH HPH ICCH

END

CROSSDIHEDRAL

# NPPC: number of cross dihedrals NOT involving H atoms in solute

0

# AP, BP, CP, DP, EP, FP, GP, HP: atom sequence numbers

# of atoms forming a dihedral

# ICC: dihedral type code

# AP BP CP DP EP FP GP HP ICC

END
